# The trajectories of Demoralization Syndrome and its related factors among elderly patients with end-stage kidney disease: a longitudinal study

**DOI:** 10.3389/fpsyt.2026.1796025

**Published:** 2026-05-18

**Authors:** Yuanyuan Yu, Xiaomei Ji, Hong Xie, Yanfang Zhang

**Affiliations:** 1 Department of Nephrology, The Second Affiliated Hospital of Bengbu Medical University, Bengbu, China; 2 School of Nursing, Bengbu Medical University, Bengbu, China; 3 School of Public Health, Bengbu Medical University, Bengbu, China

**Keywords:** chronic kidney disease, demoralization syndrome, elder, end-stage renal disease, trajectories

## Abstract

**Objective:**

Elderly ESKD patients frequently experience a range of psychosocial difficulties, with Demoralization Syndrome (DS) being especially prevalent. This study sought to delineate distinct longitudinal trajectories of DS and to examine factors associated with trajectory membership.

**Methods:**

A prospective longitudinal cohort of 363 elderly ESKD patients was recruited from Department of Nephrology in grade a hospital in Anhui, China from January 2022 to December 2025. DS was measured 4 time points from the first hemodialysis session after diagnosis to the 18 month follow-up. LCGM was applied to identify latent DS trajectory classes. Differences among classes were explored with the Two-way ANOVA, Wilcoxon rank-sum test, and multinomial logistic regression was used to assess associations between baseline characteristics and trajectory membership.

**Results:**

Three DS trajectories were identified: Severe Class (54.0%), Moderate Class (41.6%), and Mild Class (4.4%).Membership in the more adverse trajectories was significantly associated with higher use of negative coping styles, lower perceived social support, lower Barthel Index scores, and more negative perceptions of aging (all *P* < 0.05).

**Conclusions:**

Considerable heterogeneity in DS trajectories exists among elderly ESKD patients, with the majority following a severe pattern. These findings suggest that clinicians should monitor physical and cognitive functioning, regularly assess DS levels, and consider interventions targeting social support and coping strategies to mitigate worsening demoralization over time.

## 1 Introduction

Chronic kidney disease (CKD) constitutes a pressing global public health concern, with its prevalence escalating continuously and imposing a heavy medical and economic burden on society and families alike (1). Against the backdrop of the intensifying aging of the population in China, the prevalence of CKD among individuals aged 60 years and older has reached 33.0% (2). End-stage renal disease (ESRD), the terminal phase of CKD, is characterized by extremely high medical costs and exerts a devastating impact on patients’ quality of life, making it a key challenge in the prevention and control of kidney diseases. The number of ESRD patients in China has been increasing year by year, reaching 4.1405 million by the end of 2023 (3). Notably, the elderly population aged 65 years and above constitutes the predominant group of ESRD patients and bears the heaviest disease burden (4). Elderly ESRD patients are burdened with severe disease-related sequelae. They rely on long-term dialysis or kidney transplantation for survival, which is accompanied by a high complication rate, frequent hospitalizations, and substantial medical expenditures (5). Concurrently, they suffer from the deterioration of physical function as well as psychological distress including anxiety and depression, all of which severely compromise their quality of life (6, 7). Relevant studies indicate that psychological distress is prevalent among patients undergoing maintenance hemodialysis over time (8), while the proportion of psychological disorders among young patients receiving renal replacement therapy increases from 24% at baseline to 45% (9).

In recent years, increasing scholarly attention has focused on Demoralization Syndrome(DS)—a complex psychological state characterized by subjective helplessness, incapacitation to cope, and pervasive feelings of failure, representing a manifestation of existential distress (10). Current academic consensus defines demoralization as an adjustment disorder arising from perceived inability to manage stressful situations, leading to loss of control, diminished sense of purpose, and erosion of life meaning (11). Notably, the prevalence of severe DS among elderly ESRD patients in China reaches 59.4% (12). This syndrome contributes significantly to sleep disturbances, behavioral abnormalities, and reduced quality of life, adversely impacting disease management and clinical prognosis (13). Critically, DS constitutes a precursor to major depressive disorder, and in severe cases, may trigger suicidal ideation or behaviors, thereby accelerating mortality (14).

Empirical evidence indicates that dialysis duration, comorbidities, and self-care capacity significantly influence DS levels (12), potentially driving longitudinal trajectory changes in DS development. Early identification and targeted interventions for DS are critical for implementing personalized integrated management, which holds substantial importance in promoting disease recovery and enhancing quality of life. Although domestic scholars have demonstrated growing interest in DS manifestations, most current research remains confined to cross-sectional investigations, with a notable scarcity of longitudinal studies examining DS trajectories in elderly ESRD patients.

According to Rickelman’s theoretical model of DS, individuals may develop DS when facing stressors due to differential selections in coping strategies, social support, cognitive appraisals, and emotional responses (15). Therefore, this study intends to employ Latent Class Growth Modelling (LCGM) to identify trajectory patterns of DS in elderly ESRD patients, and to analyze predictive factors including coping styles, social support, the Barthel Index, and aging perception. The findings aim to provide a basis for clinically identifying key intervention groups and implementing precision psychological interventions.

## 2 Materials and methods

### 2.1 Design

This longitudinal descriptive study investigated the experiences of elderly ESRD patients. From January 2022 to December 2025, participants were consecutively recruited from the inpatient wards and outpatient clinics of the Department of Nephrology of Grade A tertiary hospitals in Anhui Province, China. Ethical approval for this study was obtained from the Bengbu Medical University Human Research Ethics Committee (Approval No. 2025-569), and according to the Declaration of Helsinki, all participants have provided informed consent forms.

### 2.2 Participants

Convenience sampling was employed. The inclusion criteria: diagnosed with ESRD and receiving maintenance dialysis treatment; aged 60 or older; clear consciousness and capable of unobstructed communication. Exclusion criteria: a history of mental illness; recent major life events such as accidents or bereavement. The estimation accuracy of the LCGM depends on the number of measurements, sample size, interclass distance, and the proportion of missing data. Under otherwise identical conditions, the sample size decreases as the number of repeated measurements increases. Specifically, a minimum sample size of 300 is required with 6 measurements (16). In the current study, given 4 repeated measurements and accounting for a 20% attrition rate, the final sample size was determined to be 400.

### 2.3 Procedures

**Figure 1:**
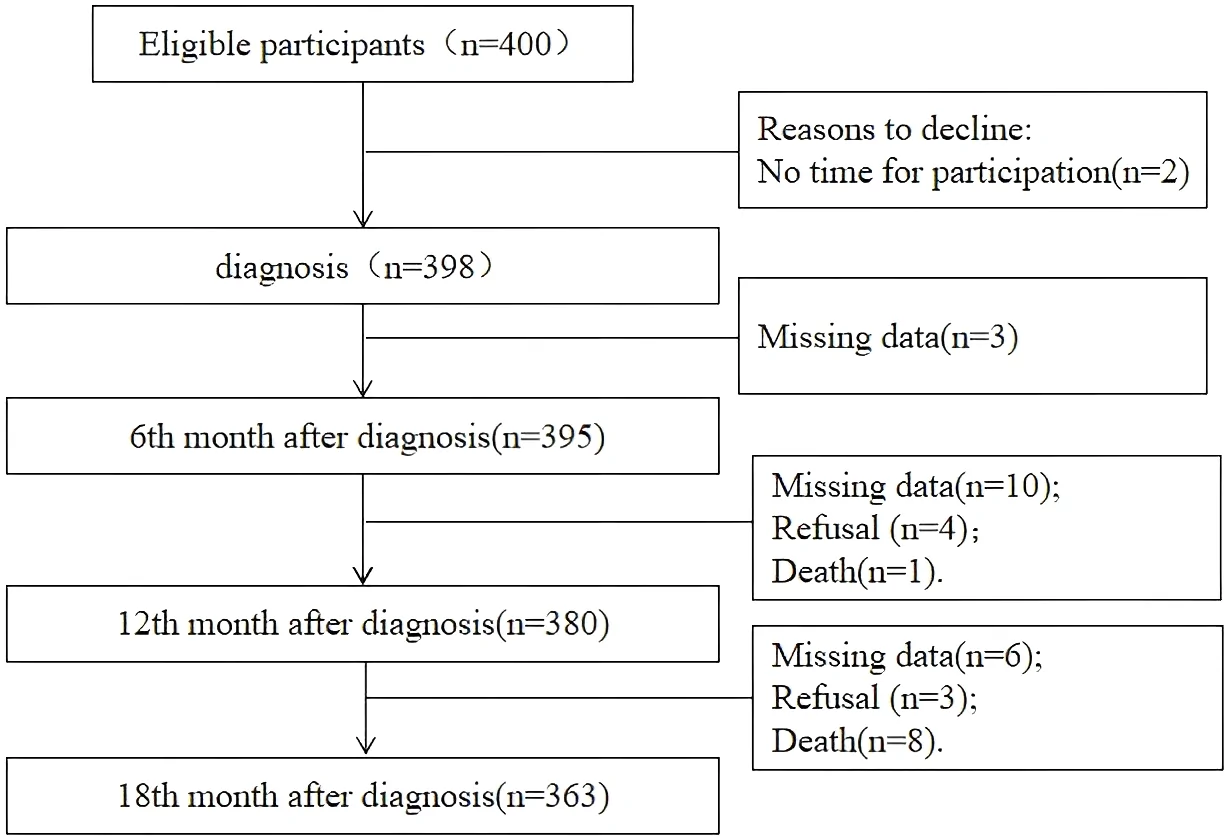
Sampling structure and attrition.

ESRD patients demonstrate progressive adaptation to their condition after 12 months of treatment (17). Furthermore, studies indicate that demoralization levels in ESRD patients escalate with prolonged dialysis duration (12). Thus, we selected 4 time points for psychological assessment: diagnosis (Time 1), 6th month after diagnosis (Time 2), 12th month after diagnosis (Time 3), 18th month after diagnosis(Time 4). Researchers collected patients data from electronic medical records to ensure the feasibility and scientific rigor of the study, as well as to reduce the rate of loss to follow-up. The uniformly trained investigator talked face-to-face with the patients and invited them to participate in the study. In T1, face-to-face questionnaires were used to collect data, including sociodemographic data, Simplified Coping Style Questionnaire, Perceived social support scale, Barthel Index, and Brief Aging Perceptions Questionnaire were collected. If the patient’s condition was poor, data was collected by phone after consultation (approximately 20 min). In the week before T2 and T4, patients were contacted via phone or WeChat to collect data. Follow-ups occurred from 10:00 to 12:00 or 14:00 to 16:00 within patients, and the schedule was negotiated with the patients if necessary. Of the 400 eligible participants, 398 completed the baseline;398, 380, an363 individuals completed the assessments 6th, 12th, and 18th months after diagnosis, respectively. Attrition rates were 0.5%, 0.7%, 3.8%, and 4.5% at each time point. Loss to follow-up was due to patient refusal to complete questionnaires, missing data, and death (**Figure 1**).

### 2.4 Measures

#### 2.4.1 Demoralization Scale

Demoralization Scale was employed to assess the level of patient demoralization (18). The questionnaire consisted of 24 questions, each rated on a scale from 0 (strongly disagree) to 4 (strongly agree), yielding total scores from 0 to 96. A score>30 indicates the presence of demoralization. In this study, Cronbach’s α for the scale ranged from 0.842 to 0.877 across the 4 time points.

#### 2.4.2 Barthel Index

Barthel Index was employed to assess patients’ ability (19). A full score of 100 indicated that the activities of daily living could be completely self-care, 61 to 99 indicated mild dysfunction, 41 to 60 indicated moderate dysfunction, 25 to 45 indicated serious dysfunction, and 0 to 40 indicated extremely serious dysfunction. In this study, Cronbach’s α for the scale was 0.716.

#### 2.4.3 Simplified Coping Style Questionnaire

Simplified Coping Style Questionnaire was employed to assess the coping styles (20). It comprises 12 items for the positive coping scale and 8 items for the negative coping scale. The scale is assessed using a 4-point Likert scale from 0 (not taken) to 3 (often taken) for each entry. A higher score on the scale suggests a greater congruence with the coping strategies typical of the assessed dimension. In this study, the scores of positive coping and negative coping scales were 0.787 and 0.731, respectively.

#### 2.4.4 Brief Aging Perceptions Questionnaire

Brief Aging perceptions questionnaire was employed to assess the attitude of the elderly toward their own aging (21). It comprises 17 items. The scale is assessed using a 5-point Likert scale from 1 (Strongly disagree) to 5 (Strongly agree), yielding total scores from 17 to 85.The higher the score, the more negative the elderly’s perception of their aging. In this study, Cronbach’s α for the scale was 0.825.

#### 2.4.5 Perceived social support scale

Perceived social support scale was employed to assess family support, friend support and others (22). The Chinese Perceived social support scale is scored on a 7-point scale ranging from 0 (none) to 7 (extremely), which comprises 14 items. The scores of 12 to 36 were considered as low support level, 37 to 60 as middle support level, 61 to 84 as high support level. In this study, Cronbach’s α were 0.955.

### 2.5 Statistical analysis

Data were analyzed using SPSS 26.0 and Mplus 8.3. The Latent Class Growth Modelling (LCGM) (23) was employed to identify the subtypes of DS trajectories. Model fit indices were used to determine the optimal number of latent classes, including the Akaike information criterion (AIC), Bayesian information criterion (BIC), adjusted BIC (aBIC), entropy, Lo–Mendell–Rubin adjusted likelihood ratio test (LMR), and bootstrapped likelihood ratio test (BLRT). The lower the statistical values of AIC, BIC, and aBIC, the better the model fit; entropy exceeded 0.8, indicating a model classification accuracy above 90%; when LRT and BLRT were statistically significant (*P* < 0.05), the Nth profile model outperformed the N-1 profile model; the optimal number of classes is thus determined.

Categorical variables are presented as frequencies and percentages, and continuous variables are expressed as means and standard deviations. Two-way ANOVA,Wilcoxon rank–sum tests were used to compare symptom scores across the 4 time points and examine demographic differences among the identified trajectory subgroups. Furthermore, multinomial logistic regression analysis was performed to evaluate baseline predictors of each DS trajectory, with statistical significance set at *P* < 0.05.

## 3 Results

### 3.1 Sample characteristics

The baseline characteristics of the study population were presented in **Table 1**.

**Table 1:** Sociodemographic and psychological variables of patients in different DS trajectories.

Variables	Total sample	Moderate class	Severe class	Mild class	*z/F*	*P*
	(n =363)	(n=151)	(n=196)	(n=16)		
Gender					11.176	0.004
Women	172	86	77	9		
Men	191	65	119	7		
Age					6.941	0.031
66~70	92	43	48	1		
60~65	116	60	51	5		
71~75	85	17	63	5		
76~80	39	17	19	3		
≥81	31	14	15	2		
Marital Status					2.769	0.250
Married	335	136	185	14		
Unmarried	28	15	11	2		
Living Alone					1.495	0.473
Yes	34	17	15	2		
No	329	134	181	14		
Number of children					1.786	0.409
≥5	39	23	14	2		
3~4	260	102	147	11		
1~2	53	21	30	2		
0	10	5	4	1		
Average Monthly Household Income (Yuan)					3.215	0.200
<1500	32	15	13	4		
1500~	173	71	95	7		
3000~	128	49	74	5		
>4500	30	16	14	0		
Education level						
Less than junior high school	81	39	37	5	2.121	0.346
Senior high school	233	90	133	10		
College or higher	49	22	26	1		
Place of residence					2.261	0.323
Urban	131	57	71	3		
Rural	232	94	125	13		
Complications,	0					
<3	128	50	69	9	3.153	0.207
3~5	164	68	91	5		
>5	71	33	36	2		
primary diseases					17.028	<0.001
renal diseases	21	1	20	0		
diabetes mellitus	50	19	29	2		
hypertension	164	64	94	6		
other etiologies	128	67	53	8		
Physical activity levels					0.506	0.776
infrequent	171	72	94	5		
occasional	114	50	55	9		
Frequent	78	29	47	2		
Dialysis modalities					28.465	<0.001
HD+HDF	56	31	18	7		
HD	207	94	106	7		
HD+HP	80	24	54	2		
HD+HDF+HP	20	2	18	0		
Positive coping		30.76 ± 4.70	29.33 ± 4.76	30.63 ± 3.81	4.106	0.017
Negative coping		10.54 ± 4.09	12.15 ± 4.60	7.44 ± 4.19	12.202	<0.001
PSSS		65.59 ± 12.26	61.60 ± 11.75	70.56 ± 13.51	7.443	0.001
BI		71.52 ± 10.44	77.83 ± 8.70	70.31 ± 11.32	20.399	<0.001
BAPQ		50.14 ± 6.68	56.29 ± 6.66	49.81 ± 5.66	39.238	<0.001

### 3.2 Identification of DS trajectories

**Figure 2:**
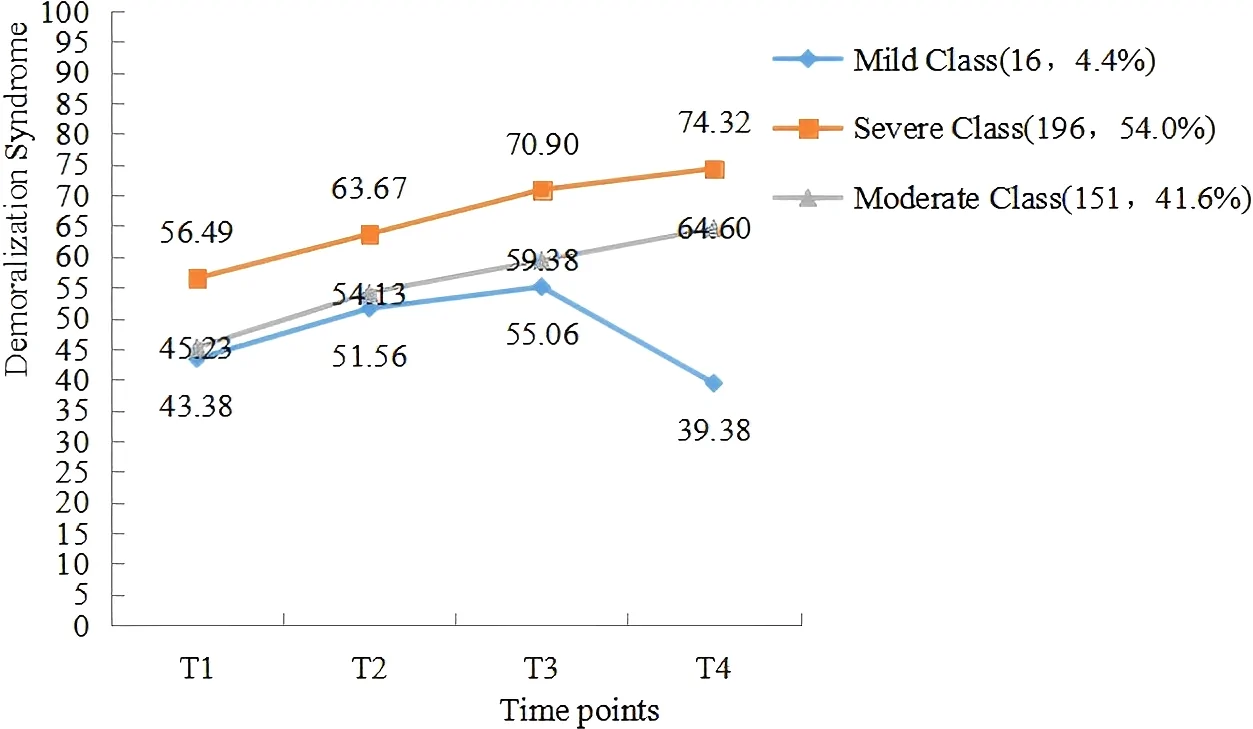
Trajectories of DS during hemodialysis period. T1: First hemodialysis after diagnosis, T2:6th month of hemodialysis, T3:12th month of hemodialysis, T4:18th month of hemodialysis.

DS scores across the 4 time points were used as the observation index, with the time parameter freely estimated, and latent categories extracted sequentially from 1 to 4. To avoid convergence at local maxima, we employed 1,000 random starts (STARTS = 1000 200) with a distinct random seed for each start and subsequently evaluated two alternative model specifications. In Model A (free estimation), the variances of the intercept and slope were estimated independently within each latent class, and residual variances were also freely estimated across classes, whereas in Model B (constrained equality) the intercept variance, slope variance, and intercept−slope covariance were constrained to be equal across all latent classes. Model fit was compared using the BIC, AIC, aBIC, and entropy; Model A yielded lower values for all information criteria (see **Table 2**) and a higher entropy, indicating clearer class separation and superior overall fit, and therefore the final analysis adopts the free estimation specification (Model A). As the number of latent categories increased from 1 to 4, AIC, BIC, aBIC, and entropy decreased, and LMR and BLRT showed statistically significant *P* values. However, when the number of categories increased from 3 to 4, LMR was not significant and entropy increased, indicating no improvement in model fit. Thus, 3 categories were retained as the optimal solution, with mean trajectories depicted in **Figure 2**.

**Table 2:** Fit statistics of LCGM with one-to-four class solutions of DS trajectories.

Model	Criterion	Parameters	AIC	BIC	aBIC	Entropy	*P* value		Class probability
							LMR	BLRT	
Model A	1	10	10919.316	10958.260	10926.534	–	–	–	1
	2	13	10896.796	10947.423	10906.180	0.881	<0.001	<0.001	0.715/0.285
	3	16	10882.265	10944.575	10893.814	0.895	0.019	<0.001	0.044/0.540/0.416
	4	19	10880.634	10954.628	10894.349	0.895	0.130	<0.001	0.057/0.519/0.023/0.401
	5	22	10883.826	10969.503	10899.707	0.757	0.205	0.105	0.377/0.061/0.003/0.019/0.540
Model B	1	7	11037.294	11064.555	11042.347	–	–	–	1
	2	10	10932.215	10971.159	10939.433	0.614	0.042	<0.001	0.942/0.058
	3	13	10913.981	10964.609	10923.365	0.633	0.003	<0.001	0.912/0.030/0.058
	4	16	10895.316	10957.626	10906.866	0.696	0.043	0.103	0.030/0.003/0.901/0.066
	5	19	10894.276	10968.269	10907.991	0.729	0.578	0.256	0.077/0.038/0.003/0.802/0.080

aBIC, adjusted Bayesian information criterion; AIC, Akaike information criterion; BIC, Bayesian information criterion; BLRT, bootstrapped likelihood ratio test; LMR, Lo-Mendell-Rubin adjusted likelihood ratio test.

**Figure 2** illustrates three latent trajectories: C1 (Moderate Class) includes 151 participants (41.6 %) with a mild baseline level (*I* = 47.748, *P* < 0.001) and a positive linear slope (*S* = 4.823, *P* < 0.05), indicating a steady increase in DS over the follow-up; C2 (Severe Class) comprises the majority of the sample (196 participants, 54.0 %) and shows a moderate baseline level (*I* = 57.164, *P* < 0.001) together with a strongly positive slope (*S* = 4.482, *P* < 0.001), reflecting rapid worsening across visits; C3 (Mild Class) contains 16 participants (4.4 %) with a mild baseline level (*I* = 48.318, *P* < 0.001) and a negative slope (*S* = −0.467, *P* < 0.001), signifying that after a brief initial rise the DS decline and remain low throughout the follow-up.

### 3.3 Predictors of DS trajectories

Significant differences in demographic and clinical characteristics were observed among the distinct DS trajectories, and the results are presented in **Table 1**. All significant variables from **Table 1** were included in a multiple logistic regression analysis. PSSS [odds ratio (OR): 1.052; 95% confidence interval (CI):1.002-1.103] were identified as protective factors for C1 compared with C2. Moreover, Negative coping (OR: 0.779; 95% CI:0.673-0.901), BI(OR: 0.940; 95% CI:0.887-0.997) and BAPQ(OR: 0.895; 95% CI:0.816-0.982) were identified as risk factors for C1 compared with C2. PSSS [OR: 1.020; 95% CI:0.999-1.041] were identified as protective factors for C3 compared with C2. Moreover, Negative coping (OR: 0.939; 95% CI:0.888-0.993), BI(OR:0.959; 95% CI:0.933-0.986) and BAPQ(OR: 0.883; 95% CI:0.845-0.924) were identified as risk factors for C3 compared with C2 (**Table 3**).

**Table 3:** Multiple progressive logistic regression^a^ predicting DS trajectories.

Variables (reference)	C1 vs C2					C3vs C2				
	B	SE	*Wald χ^2^*	*P*	OR(95%CI)	B	SE	*Wald χ^2^*	*P*	OR(95%CI)
Negative coping	-0.250	0.075	11.274	0.001	0.779(0.673,0.901)	-0.063	0.029	4.862	0.027	0.939(0.888,0.993)
BI	-0.061	0.030	4.214	0.040	0.940(0.887,0.997)	-0.041	0.014	8.540	0.003	0.959(0.933,0.986)
BAPQ	-0.111	0.047	5.520	0.019	0.895(0.816,0.982)	-0.124	0.023	29.215	<0.001	0.883(0.845,0.924)
PSSS	0.050	0.024	4.239	0.040	1.052(1.002,1.103)	0.020	0.010	3.537	0.060	1.020(0.999,1.041)

C1, Moderate Class; C2, Severe Class; C3, Mild Class.

## 4 Discussion

The novelty of this longitudinal study lies in its identification of heterogeneous DS trajectories among patients with ESKD in the elderly. Three distinct trajectory groups were identified: Severe Class, Moderate Class, and Mild Class. Approximately 54.0% of the sample (Severe Class) reported severe levels of DS at diagnosis, with a tendency for symptom progression over time. This may be due to the psychological impact of being compelled to accept maintenance hemodialysis as the life-sustaining intervention likely precipitates a sharp elevation in DS levels (24). Notably, while maintenance hemodialysis effectively removes metabolic waste and maintains electrolyte balance, it fails to fully compensate for lost renal function (25). Prolonged dialysis duration leads to the accumulation of uremic toxins, which subsequently induce a progressively worsening cluster of clinical symptoms, including fatigue, pruritus, and arthralgia (26). This escalating symptom burden significantly heightens the risk of depression (25), consequently impairing quality of life (26). Furthermore, the demanding treatment regimen—requiring thrice-weekly sessions, each lasting approximately four hours—imposes considerable physical and mental exhaustion on both patients and caregivers due to the necessity for frequent hospital visits. Coupled with the substantial financial burden associated with ongoing medical costs, these multifaceted stressors exacerbate the challenges of disease management, fostering feelings of despair and helplessness among patients (27). This study observed that 41.6% of patients were categorized as the “Moderate Class”. Although initial dialysis can alleviate clinical discomfort symptoms by removing toxins, thereby enhancing patient acceptance of treatment, the levels of DS did not improve with increasing treatment sessions. This may be associated with the patient cohort’s overall advanced age, high comorbidity burden, suboptimal dialysis efficiency, physical frailty, and fatigue. Symptoms such as fatigue, weakness, and diminished physical capacity can lead to restricted physical activity in these patients (28). Concurrently, prolonged physical frailty can exacerbate negative emotions, including feelings of helplessness, depression, and anxiety (29). The observation that only 4.4% of patients were categorized in the “Mild Class” indicates that DS peaks at the 12th month at diagnosis in elderly ESKD patients, followed by a progressive reduction with prolonged dialysis duration. This pattern suggests significant physical and psychological distress at the initial diagnosis of uremia, with subsequent physiological and psychological adaptation to the regular dialysis regimen over time.

The findings of this study demonstrate that higher levels of negative coping are associated with elevated DS levels. This correlation may be attributed to the protracted nature of ESKD, where an indefinite treatment trajectory predisposes patients to adopt passive attitudes toward daily challenges (30). However, extant research indicates that avoiding the existence of stressors or evading an appraisal of their severity can effectively mitigate psychological pressure, yielding beneficial effects on both mental health and treatment adherence (31). Consequently, healthcare professionals should refrain from simplistically interpreting negative coping as mere escapism. Instead, it ought to be conceptualized as a mechanism for attentional diversion or transient conflict resolution. Higher levels of social support are associated with lower levels of DS. Patients undergoing ESKD endure prolonged physical and psychological suffering due to the incurable nature of their condition, which impedes their ability to fulfill familial and social responsibilities and imposes a significant caregiving burden. Concurrently, complications, adverse treatment effects, restrictions on diet and physical activity, and financial pressures predispose patients to social avoidance behaviors (32). These behaviors further diminish their social support, ultimately perpetuating high levels of DS. This study revealed that higher levels of aging perceptions were associated with elevated DS levels. Conversely, a higher Barthel Index correlated with lower DS levels. Aging serves as a significant predictor of cognitive function (33). Individuals with positive aging perceptions exhibit greater confidence in engaging in cognitively stimulating activities to maintain cognitive health; conversely, those with negative aging perceptions often hold pessimistic attitudes toward their own capabilities and aging expectations, leading to reduced motivation for social or cognitive activities and a consequently higher risk of cognitive impairment (34). The Barthel Index, which assesses activities of daily living, reflects functional independence (35). Impairment in activities of daily living significantly diminishes the quality of life among elderly patients, adversely impacts their psychological well-being, and consequently exacerbates DS.

### 4.1 Study limitations

There are several limitations in this study. First, the current study utilized a convenience sampling method exclusively from Anhui Province, China, resulting in limited representativeness. Future research should implement multi-center, large-scale studies to enhance generalizability and scientific rigor. Additionally, the fixed time-point assessment design meant that the dynamic fluctuations of DS (disharmony syndrome) among elderly ESKD patients were not fully captured. A longer follow-up period, including regular follow-up assessments at 2, 3 years and beyond, is suggested in subsequent research. The study did not include some clinically relevant variables, specifically, dialysis adequacy, electrolyte imbalances, inflammatory markers, and formal cognitive/affective assessments were unavailable. While these variables are associated with our exposure and outcome, their omission does not invalidate our main findings, though residual confounding cannot be fully ruled out (36). To enhance our findings’ robustness, future studies may use sensitivity analysis (e.g., E-value) to quantify unmeasured confounding impact (37). Incorporating more comprehensive clinical covariates will also help reduce this bias. Since variables like coping styles, social support, Barthel Index, and aging perception may change over time, future longitudinal tracking with concurrent collection of these variables will better elucidate their relationship with DS and supplement our findings.

### 4.2 Clinical implications

Despite limitations, this study identified negative coping styles, social support, the Barthel Index, and perception of aging as key factors influencing DS trajectories. Clinicians should pay special attention to the physical and cognitive functions of patients, evaluate the dynamic DS level of elderly ESKD patients based on the evaluation results, starting from social support and coping methods, and take timely intervention measures.

## 5 Conclusions

This study elucidated DS trajectory groups in the elderly ESKD patients. Three distinct trajectory groups were identified: Severe Class, Moderate Class, and Mild Class—demonstrating heterogeneity in DS at diagnosis to 18th month after diagnosis. Longitudinal analysis identified negative coping styles, social support, the Barthel Index, and perception of aging as key factors influencing DS trajectories. These factors should be considered by healthcare professionals to deliver appropriate psychosocial care. The findings further suggest potential implications for future identification of the elderly ESKD patients in need of targeted interventions based on their DS trajectory patterns.

## Data Availability

The data were not publicly available due to privacy and ethical restrictions. Requests to access the datasets should be directed to Yanfang Zhang. Email: 1696545174@qq.com.
